# Huangqi Decoction Alleviates Alpha-Naphthylisothiocyanate Induced Intrahepatic Cholestasis by Reversing Disordered Bile Acid and Glutathione Homeostasis in Mice

**DOI:** 10.3389/fphar.2017.00938

**Published:** 2017-12-21

**Authors:** Jia-Sheng Wu, Yi-Fei Li, Yuan-Yuan Li, Yan Dai, Wen-Kai Li, Min Zheng, Zheng-Chun Shi, Rong Shi, Tian-Ming Wang, Bing-Liang Ma, Ping Liu, Yue-Ming Ma

**Affiliations:** ^1^Department of Pharmacology, School of Pharmacy, Shanghai University of Traditional Chinese Medicine, Shanghai, China; ^2^Key Laboratory of Liver and Kidney Diseases (Ministry of Education), Institute of Liver Diseases, Shuguang Hospital, Shanghai University of Traditional Chinese Medicine, Shanghai, China; ^3^Shanghai Key Laboratory of Compound Chinese Medicines, Shanghai University of Traditional Chinese Medicine, Shanghai, China

**Keywords:** intrahepatic cholestasis, Huangqi decoction, traditional Chinese medicine, Nrf2, metabolomics

## Abstract

Intrahepatic cholestasis is a serious symptom of liver disorders with limited therapies. In this study, we investigated the efficacy of Huangqi decoction (HQD), a two-herb classic traditional Chinese medicine (TCM), in the treatment of alpha-naphthylisothiocyanate (ANIT)-induced intrahepatic cholestasis in mice. HQD treatment ameliorated impaired hepatic function and tissue damage. A metabolomics study revealed that the endogenous metabolites significantly affected by HQD were related to bile acid (BA) biosynthesis and glutathione metabolism pathways. HQD treatment decreased the intrahepatic accumulation of cytotoxic BAs, normalized serum BA levels, and increased biliary and urinary BA excretion. Additionally, HQD restored the hepatic glutathione content and suppressed reactive oxygen species (ROS) in cholestatic mice. Protein and gene analysis revealed that HQD increased the expression of the hepatic metabolizing enzymes cytochrome P450 (CYP) 2B10 and UDP glucuronosyltransferase family 1 member A1 (UGT1A1), as well as multidrug resistance-associated protein 2 (Mrp2), Mrp3, and Mrp4, which play crucial roles in BA homeostasis. Further, HQD increased the protein expression of glutamate-cysteine ligase, which is involved in the synthesis of glutathione. Importantly, HQD increased the nuclear expression of nuclear factor-E2-related factor-2 (Nrf2). In conclusion, HQD protects against intrahepatic cholestasis by reversing the disordered homeostasis of BAs and glutathione.

## Introduction

Intrahepatic cholestasis is a clinical syndrome characterized by systemic and intrahepatic retention of excessive toxic bile acids (BAs). It is one of the most common symptoms of several acquired and hereditary liver diseases such as hepatitis (Kang et al., 2015), drug-induced liver injury (Liu et al., 2013), pregnancy (Reyes, 2015), and primary biliary cirrhosis (Srivastava, 2014). Improper treatment of this condition may lead to the development of fibrosis, cirrhosis, and eventual liver failure. Currently, ursodeoxycholic acid is widely used for cholestatic liver disease, although it has only short-term or modest beneficial effects or both (Ali et al., 2016). Therefore, novel therapeutic approaches are needed.

BAs are the main endogenous substances synthesized by hepatocytes, and are subsequently secreted into the bile canaliculus. The detergent-like property of BAs may cause apoptosis and necrosis at high concentrations (Marin et al., 2015); therefore, hepatic BA levels must be tightly regulated. Numerous metabolizing enzymes and transporters play key roles in maintaining BA homeostasis (Dietrich and Geier, 2014). Dysfunction or abnormal expression of these enzymes and transporters may disturb bile flow and induce hepatic cholestasis (Trauner and Boyer, 2003; Salvatore et al., 2012; Cai et al., 2014; Cuperus et al., 2014; Chai et al., 2015; Meng et al., 2015). Nuclear receptors and transcription factors such as pregnane X receptor (PXR), farnesoid X receptor (FXR), constitutive androstane receptor (CAR), and nuclear factor-E2-related factor-2 (Nrf2), which regulate these genes, may be potential therapeutic targets in cholestasis (Weerachayaphorn et al., 2012; Chen et al., 2014).

For millennia, herbs and traditional medicines have been used globally for the prevention and treatment of diseases, and are sources of numerous prescription and new drugs (Dhiman and Chawla, 2005; Chen et al., 2014; Ji et al., 2015; Pan et al., 2015; Pang et al., 2015). Radix astragali (*Astragalus membranaceus* [Fisch.]) Bge., root) and radix glycyrrhizae (*Glycyrrhiza uralensis* Fisch., root and rhizome) are two popular herbal medicines. Both are routinely used in China to enhance hepatic function (Qi et al., 2013; Shahzad et al., 2015; Jung et al., 2016). Huangqi decoction (HQD), a classic traditional Chinese medicine (TCM), consists of radix astragali and radix glycyrrhizae and is used for consumptive disease and chronic liver diseases since the Song Dynasty. In our previous preclinical studies, HQD reduced the liver injury caused by dimethylnitrosamine and decreased the liver fibrosis induced by bile duct ligation (Du et al., 2012; Liu et al., 2012; Li et al., 2015; Zhang G. B. et al., 2015).

However, whether HQD has a protective effect against intrahepatic cholestasis and any potential underlying mechanism are still unknown. Alpha-naphthylisothiocyanate (ANIT) is a chemical that is widely used to mimic human intrahepatic cholestasis. ANIT can selectively damage the bile duct epithelial cells and causes cholangitis and subsequent intrahepatic cholestasis (Dietrich et al., 2001). In the present study, we investigated the effect of HQD on intrahepatic cholestasis in an ANIT-induced mouse model of intrahepatic cholestasis and explored the potential underlying therapeutic mechanism using a metabolomics assay coupled with pathway analysis.

## Materials and methods

### Chemical compounds and reagents

ANIT, carbamazepine, mycophenolic acid, cholic acid (CA), glycocholic acid (GCA), taurocholic acid (TCA), chenodeoxycholic acid (CDCA), glycochenodeoxycholic acid (GCDCA), taurochenodeoxycholic acid (TCDCA), deoxycholic acid (DCA), glycodeoxycholic acid (GDCA), taurodeoxycholic acid (TDCA), ursodeoxycholic acid (UDCA), glycoursodeoxycholic acid (GUDCA), tauroursodeoxycholic acid (TUDCA), lithocholic acid (LCA), glycolithocholic acid (GLCA), taurolithocholic acid (TLCA), hyodeoxycholic acid (HDCA), glycohyodeoxycholic acid (GHDCA), and taurohyodeoxycholic acid (THDCA) were purchased from Sigma-Aldrich (St. Louis, MO, USA). Chromatography grade acetonitrile was purchased from Merck (Darmstadt, Germany). Water was purified using a MilliQ water system (Millipore, Bedford, MA, USA). Chromatography grade acetic acid, formic acid, and methanol were provided by Tedia Company (Fairfield, OH, USA).

### Preparation and ultra-pressure liquid chromatography coupled with mass spectrometry (UPLC-MS) analysis of HQD

HQD consists of Radix astragali (*A. membranceus* [Fisch]) Bge. root) and Radix glycyrrhizae (*G. uralensis* Fisch, root and rhizome) in a 6:1 ratio. HQD extract powder (1.2 g equivalent to 6 g of Radix astragali crude herbs and 1 g of Radix Glycyrrhizae crude herbs) was prepared by Jiangyin Tianjiang Pharmaceutical Co., Ltd. (Jiangsu, China), using high-quality herbs. Briefly, Radix astragali and Radix glycyrrhizae were mixed at a ratio of 6:1 and then extracted with boiling water, and the aqueous extract were vacuum-dried (60°C) to obtain the powdered extract. The content of the major compounds of the HQD extract powder was determined using ultra-performance liquid chromatography (UPHLC)-mass spectrometry (MS) using standardized chemicals as described previously (Luo et al., 2014).

### Animals and treatments

Male, 8-week-old C57BL/6 mice were purchased from Shanghai Laboratory Animal Center of Chinese Academy of Science (Shanghai, China), and were maintained in a room at 22–24°C with a 12-h light/dark cycle and 55–60% relative humidity. The mice had access to standard rodent food and water *ad libitum*. All animal experimental procedures were approved by the Committee on the Use of Live Animals for Teaching and Research of the Shanghai University of Traditional Chinese Medicine (Approval Number: 20150401), and all experiments were performed according to the guideline of this committee. The animal experimental design is shown in Table 1. Sixty mice were randomly assigned to six groups: Vehicle, Vehicle + HQD 2 g/kg, ANIT + Vehicle, ANIT + HQD 1 g/kg, ANIT + HQD 2 g/kg, ANIT + UDCA. HQD extract powder or UDCA was intragastrically (i.g) administered to mice at a volume of 10 mL/kg body weight once daily for 14 days. The Vehicle and ANIT groups were administered equal amounts of distilled water. ANIT (dissolved in corn oil, 50 mg/kg, i.g.) was administered to mice on day 12 day after pretreatment with HQD or UDCA. Then, 48 h after ANIT treatment, all the mice were sacrificed, and the blood was collected from the eye venous plexus. The liver samples were collected and divided into three parts: one part was fixed with 4% paraformaldehyde in phosphate buffer, another part was immersed in 2.5% glutaraldehyde in 0.1 M cacodylate buffer (pH 7.4), and the third part was snap-frozen in liquid nitrogen, and stored at −80°C until use. The gallbladder of each mouse was harvested, and the bile was subsequently collected.

**Table 1:** Animal experimental design.

**Group**	***n***	**Treatment**	**Drug dose (mg/kg or g/kg)**	**Administration volume (ml/kg bodyweight)**
Vehicle	10	i.g. distilled water for 14 days	–	10
HQD 2g/kg	10	i.g HQD for 14 days	2 g/kg	10
ANIT+Vehicle	10	i.g. distilled water for 14 days, i.g. ANIT on day 12	–	10
ANIT + HQD 1g/kg	10	i.g. HQD for 14days, i.g. ANIT on day 12	1 g/kg	10
ANIT + HQD 2g/kg	10	i.g. HQD for 14days, i.g. ANIT on day 12	2 g/kg	10
ANIT + UDCA	10	i.g. UDCA for 14days, i.g. ANIT on day 12	100 mg/kg	10

*HQD, Huangqi decoction; ANIT, alpha-naphthylisothiocyanate; i.g: intragastrically (i.g) administration*.

### Serum biochemistry and histologic analysis

The dissected liver tissues were immediately fixed in formalin, paraffin-embedded, sectioned, and stained with hematoxylin and eosin (H&E) following a standard protocol. The H&E-stained liver sections were examined using an Olympus BX41 microscope (Olympus Corporation; Tokyo, Japan). The serum alanine aminotransferase (ALT), aspartate aminotransferase (AST), and alkaline phosphatase (ALP) activity, as well as the serum total bile acid (TBA) and bilirubin levels, were measured on a Cobas Integra 400 Clinical Analyzer (Roche Diagnostics).

### Electron microscopy

The liver was fixed in 2.5% glutaraldehyde (0.2 M cacodylate buffer, pH 7.4) and embedded in epoxy resin. Ultrathin sections were double-stained with 1.25% uranium acetate and 0.4% lead citrate and were examined using a JEM100CX-α electron microscope (JEOL, Ltd., Tokyo, Japan).

### Serum metabolomics study

#### Sample preparation

A 20-μL serum aliquot was deproteinized with 100 μL acetonitrile containing the internal standard (IS; positive: carbamazepine 20 ng/mL, negative: mycophenolic acid 320 ng/mL). The sample mixture was vortexed for 1 min and centrifuged at 16,000 rpm for 10 min at 4°C. The quality control (QC) samples were prepared by combining equal aliquots of all serum samples that were processed according to standard serum sample preparation (Huang et al., 2013; Liu et al., 2017). The QC samples were analyzed before, during, and after each analytical run.

#### Instrumentation and operation conditions

The LC separation was performed using a UPLC system (Dionex, Thermo Fisher Scientific; Sunnyvale, CA, USA). The analytical column was a Waters ACQUITY UPLC BEH C18 column (2.1 × 100 mm, 1.7 μm) (Waters, Co., Milford, MA, USA). The column and automatic sampler were maintained at 35 and 4°C, respectively and the injection volume was 5 μL. The gradient elution was run for 20 min at a flow rate of 0.3 mL/min with mobile phases consisting of water with 0.1% formic acid (A) and acetonitrile with 0.1% formic acid (B).

The elution was run on the following schedule: B, 10% from 0.1 to 2 min, and linearly increased from 10 to 40% within 5 min; B, increased linearly from 40 to 80% within 4 min; B, increased linearly from 80 to 90% within 4 min; and B, maintained at 90% for 0.5 min. At 15.5 min, B was adjusted to 10% and was equilibrated for 4.5 min. The UPLC system was connected to a linear trap quadrupole (LTQ)-orbitrap elite MS system (Thermo Fisher Scientific, Bremen, Germany) using a heated electrospray ionization (ESI) source in both positive and negative ionization modes. The MS parameters were as follows: ion spray voltage, 3.8 kV(+) and 3.2 kV(−); capillary and heater temperature, both 350°C; sheath and auxiliary gas flow rate, 45 and 15 psi; and S-Lens RF level, 60%.

#### Data processing and standardization of metabolites, identification of potential biomarkers, and metabolic pathway analysis

Data preprocessing and analysis, identification of potential biomarkers, and pathway analysis were performed as previously described (Huang et al., 2013).

### Bile acid profiling measured using UPLC-tandem MS (MS/MS)

The profiles of the hydrophilic and hydrophobic BAs in the serum, liver, and bile of mice in each group were measured using previously published UPLC-MS/MS methods (Yang et al., 2008) with slight modifications. The sample preparation, chromatography, and MS conditions are described in detail in the Supplementary Methods. Calibration curves are presented in Supplementary Tables 1–3.

### Analysis of hepatic glutathione content

Glutathione was determined using a commercially available glutathione assay kit (Cayman Chemicals, Ann Arbor, MI, USA). Briefly, 20 mg of the liver tissue samples was weighed, homogenized with 0.1 M sodium phosphate buffer (pH 7.4), and the homogenates were subsequently centrifuged with metaphosphoric acid (MPA) solution to remove the proteins. A 50-μL aliquot of the homogenate was mixed with 150 μL reaction buffer (provided in the kit), vortexed, and the absorbance was read at 405 nm within 30 min. The glutathione content was calculated using a standard solution of glutathione.

### Measurement of hepatic reactive oxygen species (ROS) content

The liver reactive oxygen species (ROS) levels were measured as previous study reported (Wang et al., 2017). The mouse liver tissue was homogenized with 20-fold volume of ice cold 1 × PBS and then centrifuged (120,000 × g, 10 min, 4°C). The protein concentrations in the supernatants were determined using bicinchoninic acid (BCA) kits (Beyotime Biotechnology, Jiangsu, China). The ROS in the supernatant were determined with ELISA kits for mouse ROS (Weitong, Shanghai, China) according to the manufacturer's protocol. The optical density was detected at 450 nm with a microplate reader (BioTek Instruments, Vermont, USA).

### Quantitative real-time polymerase chain reaction (qPCR) analysis

Total RNA was extracted from 20 mg liver samples using Trizol, and the cDNA was subsequently synthesized using the SuperScript cDNA synthesis kit, and the quantitative real-time polymerase chain reaction (qPCR) was performed using the ABI-StepOnePlus sequence detection system (Applied Biosystems, CA, USA) using the Fast SYBR Green mix kit. The primers used are presented in Supplementary Table 4. The relative mRNA expression levels of the detected genes (Supplementary Table 4) were calculated using the 2^−ΔΔCt^ method. The expression of glyceraldehyde 3-phosphate dehydrogenase (*GAPDH*) mRNA was used as the endogenous reference control (Wu et al., 2014).

### Western blot analysis

The liver samples (30 mg) were homogenized in a radioimmunoprecipitation assay buffer. The lysate proteins were separated using sodium dodecyl sulfate-polyacrylamide gel electrophoresis (SDS-PAGE) and were electro-blotted onto nitrocellulose membranes. The membranes were blocked for 1 h in 5% non-fat milk, and then incubated overnight with primary antibodies against Mrp2, Mrp3, Mrp4 (Santa Cruz Biotechnologies, Inc., Santa Cruz, CA, USA), glutamate-cysteine ligase, catalytic subunit (GCLC), glutamate-cysteine ligase modifier subunit (GCLM), heme oxygenase-1 (HO-1), Nrf2, extracellular signal-regulated kinase 1/2 (ERK1/2), p38, phosphorylated (phospho)-ERK1/2 or phospho-p38 (Cell Signaling Technology, Danvers, MA, USA) at 4°C. The membranes were subsequently washed with Tris-buffered saline/0.1% (v/v) Tween-20 and were incubated for 1 h with secondary antibodies. After washing, the protein bands were detected using the FluorChem E image detection system (ProteinSimple, San Jose, CA, USA). GAPDH, β-actin, or Lamin A/B was used as a loading control.

### Immunohistochemistry

After the mice were euthanized, their livers were immediately removed and fixed in 10% neutral-buffered paraformaldehyde at 4°C. Selected tissue samples were embedded in paraffin, sectioned, and stained with an Mrp2, Mrp4, or Nrf2 primary antibody. The sections were mounted with DPX mountant (Sigma-Aldrich, St Louis, MO, USA) for histological analysis. A terminal deoxynucleotidyl transferase-mediated dUTP nick-end-labeling assay was performed using a commercially available kit (Chemicon, Temecula, CA, USA) following the manufacturer's instructions.

### Statistical analyses

The data are expressed as the means ± standard deviation (*SD*). A one-way analysis of variance and the Student-Newman-Keul (SNK) test were used to analyze the differences between groups, and a *p* < 0.05 was considered statistically significant while *p* < 0.01 was considered highly significant.

## Results

### HQD protects against intrahepatic cholestasis-induced liver injury

**Figure 1:**
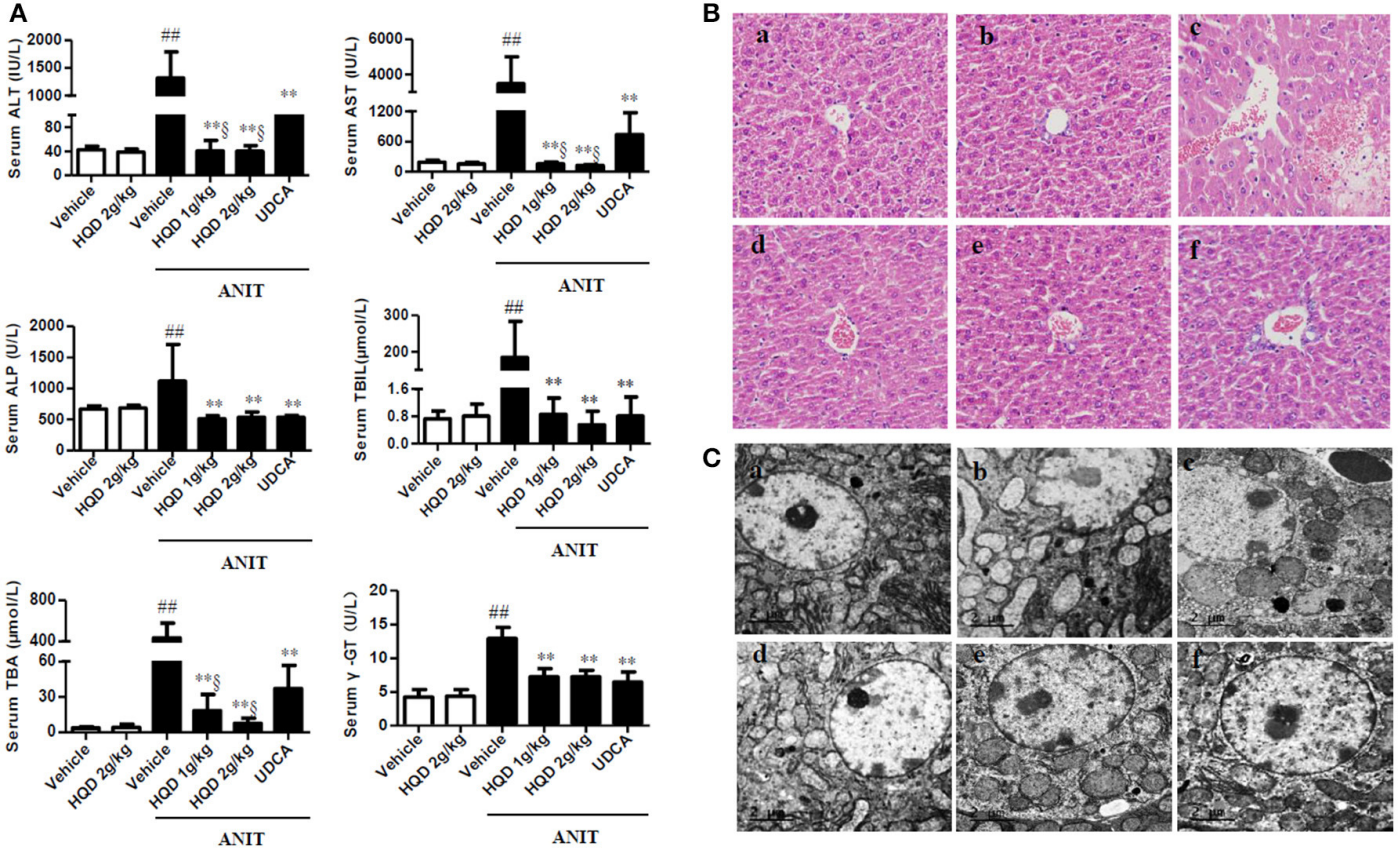
Huangqi decoction (HQD) protects against alpha-naphthylisothiocyanate (ANIT)-induced intrahepatic cholestasis in mice. **(A)** Serum biochemical parameters **(B)** Liver morphology was observed using light microscopy (original magnification, ×200) and **(C)** microstructure was observed using electron microscopy (scale bar, 2 μm, original magnification × 6,000). Groups: a, Vehicle; b, HQD 2 g/kg; c, ANIT + Vehicle; d, ANIT + HQD 1 g/kg; e, ANIT + HQD 2 g/kg; f: ANIT + UDCA. Data are means ± *SD, n* = 10 for serum biochemical parameters and light microscopy and *n* = 3 for electron microscopy; ^##^*p* < 0.01 and ^**^p < 0.01 compared with Vehicle and ANIT + Vehicle groups; ^§^*p* < 0.01 compared with the UDCA group. ANIT, alpha-naphthylisothiocyanate; ALT, alanine aminotransferase; AST, aspartate transaminase; ALP, alkaline phosphatase; TBA, total bile acid; TBIL, total bilirubin. γ-GT, γ-glutamyl transpeptidase.

Serum ALT and AST, biochemical indicators of liver damage, significantly increased in the ANIT + vehicle group compared with that in the vehicle group. However, low- and high-dose HQD pretreatments markedly reduced the increases in serum ALT and AST (Figure 1A). Serum ALP and gamma-glutamyl transferase (γ-GT) levels, which are biochemical indicators of biliary toxicity, increased in the ANIT + vehicle group and were significantly reduced by the HQD pretreatments (Figure 1A). Additionally, both low- and high-dose HQD pretreatment markedly reversed the ANIT-induced increase in total serum bilirubin and total BAs (Figure 1A). HQD treatment alone did not affect liver function in normal mice. Moreover, HQD treatments significantly decreased the ANIT-induced increase in liver weight of cholestatic mice but did not affect the body weight of mice with cholestasis (Supplementary Figure 1).

The H&E-stained liver tissue of the vehicle group exhibited a normal structure with no abnormal morphological changes, whereas that of the group treated with ANIT alone showed acute inflammatory cell infiltration, edema, and hepatic necrosis (Figure 1B). In comparison, the H&E-stained liver tissue of the HQD-pretreated (both low- and high-dose) groups exhibited a mild degree of hydropic hepatocyte degeneration with less inflammatory cell infiltration (Figure 1B), highly similar to what was observed in the vehicle group. Moreover, electron microscopy analysis revealed an abnormal dilatation of the endoplasmic reticulum in hepatocytes of the group treated with ANIT alone, which was significantly ameliorated by HQD pretreatment (Figure 1C).

### Metabolomics study of effects of HQD in cholestatic mice

#### Data acquisition

**Figure 2:**
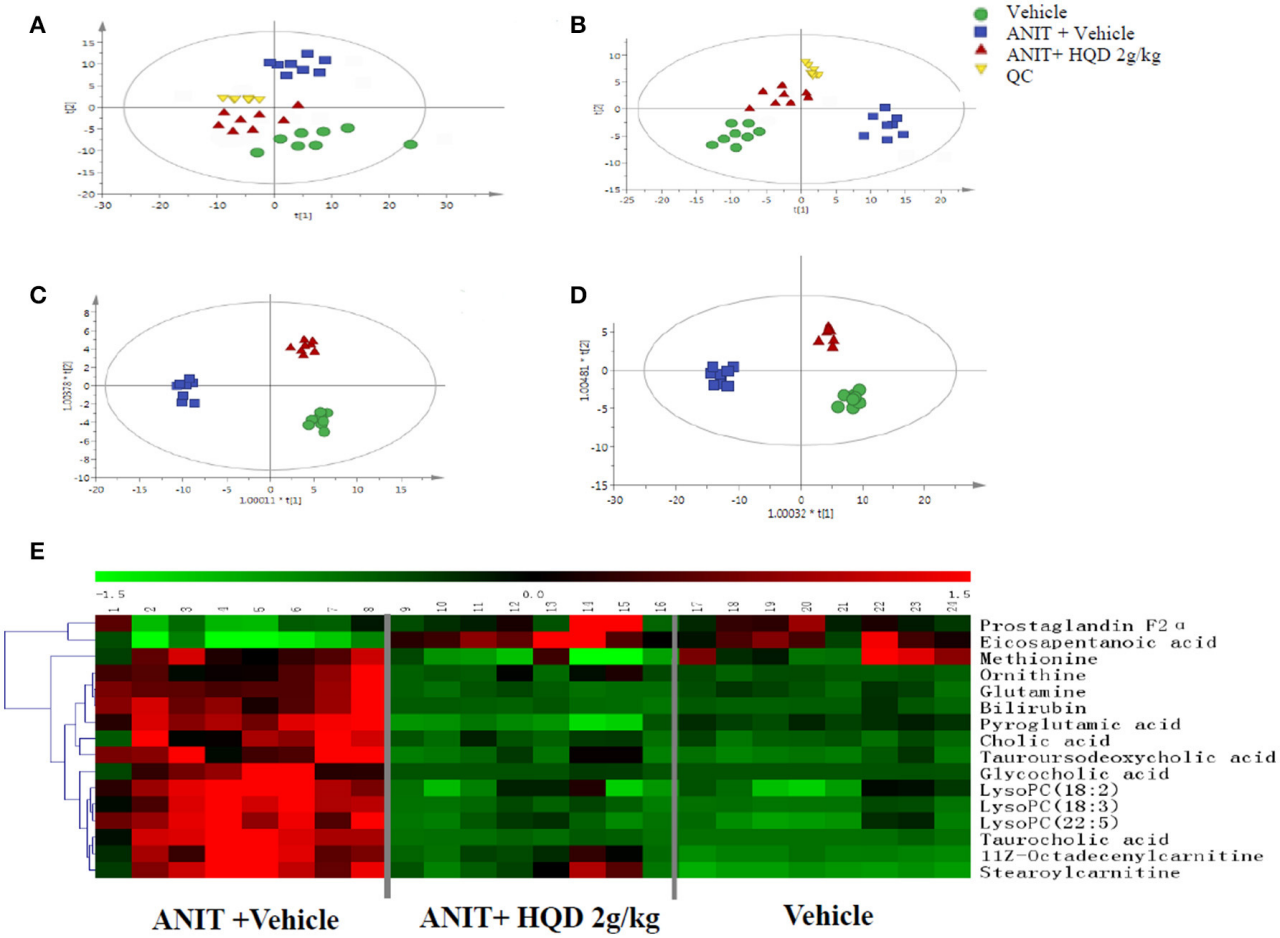
Metabolomics study of Huangqi decoction (HQD) in mice with intrahepatic cholestasis. Principal components analysis (PCA) score plot in **(A)** positive ion mode, R^2^X = 0.974, *Q*^2^ = 0.811 and **(B)** negative ion mode, R^2^X = 0.963, Q^2^ = 0.798. Orthogonal partial least squares discriminant analysis (OPLS-DA) score plot in **(C)** positive ion mode, R^2^X = 0.515, R^2^Y = 0.996, Q^2^ = 0.993 and **(D)** negative ion mode, R^2^X = 0.356, R^2^Y = 0.996, Q^2^ = 0.955. **(E)** Hierarchical clustering heat map of metabolites with different levels in vehicle, ANIT + Vehicle, and ANIT + HQD 2 g/kg groups. ANIT, alpha-naphthylisothiocyanate; QC, quality control.

The QC samples were tightly clustered in both the positive and negative ionization mode, illustrating the stability of the UPLC/MS method throughout the entire run [Figures 2A,B, showing principal components analysis (PCA) plot]. Further, a supervised orthogonal partial least squares discriminant analysis (OPLS-DA) was used to divide the vehicle, ANIT + vehicle (Supplementary Figure 2), ANIT + vehicle, and ANIT+ HQD 2 g/kg (Supplementary Figure 3), groups. It was also used to facilitate the screening of potential marker metabolites. After screening using the “VIP > 1.00” and “*p* < 0.05” settings, we obtained 215 (ESI+) and 320 (ESI−) metabolite variables in the vehicle group vs. the ANIT + vehicle group, and 209 (ESI+) and 338 (ESI−) metabolite variables in the ANIT + vehicle group vs. the ANIT + HQD 2 g/kg group for further identification. The results of the OPLS-DA showed adequate separation of the data pertaining to the three groups (Figures 2C,D). The model statistics R^2^X, R^2^Y, and Q^2^ indicated that the models were robust and not the result of statistical overfitting.

#### Identification of potential biomarkers

The differences in the metabolites identified by comparing various pairs of groups are presented in Supplementary Table 5. Compared with the normal control group, 30 serum metabolites showed significantly different levels in the ANIT + vehicle group, and 16 of these differences were reversed by HQD treatment (Table 2). Twelve of the 16 differentially expressed metabolites were identified based on comparisons with authenticated standards, and the others were deduced using accurate molecular weights, MS/MS fragments, and metabolomics databases. Figure 2E illustrates the changes in these potential biomarkers in different groups as a heat map.

**Table 2:** Endogenous metabolites identified in the serum of mice included in this study.

**No**.	***t*_R_ (min)**	**Molecular ion**	**Compound MW**	**Measured Mass (Da)**	**VIP**	**Formula**	**Metabolites**	**HMDB**
M1	0.78	[M+H]^+^	132.0893	133.0965	2.0105	C_5_H_12_N_2_O_2_	Ornithine^*^	00214
M2	0.84	[M-H]^−^	146.0694	145.0621	1.2415	C_5_H_10_N_2_O_3_	Glutamine^*^	00641
M3	0.95	[M+H]^+^	149.0503	150.0576	2.0454	C_5_H_11_NO_2_S	Methionine^*^	00696
M4	0.97	[M+OH]^−^	129.0427	146.0457	1.2672	C_5_H_7_NO_3_	Pyroglutamic acid^*^	00267
M5	0.97	[M+H]^+^	129.0422	130.0494	1.9262	C_5_H_7_NO_3_	Pyroglutamic acid^*^	00267
M6	3.77	[M+H]^+^	515.2887	516.2960	4.1002	C_26_H_45_NO_7_S	Taurocholic acid^*^	00036
M7	3.99	[M-H]^−^	499.2952	498.2894	2.1877	C_26_H_45_NO_6_S	Tauroursodeoxycholic acid^*^	00874
M8	4.58	[M-H]^−^	408.2865	407.2795	2.0064	C_24_H_40_O_5_	Cholic acid^*^	00619
M9	4.84	[M-H]^−^	465.3075	464.3009	3.2533	C_26_H_43_NO_6_	Glycocholic acid^*^	00138
M10	4.93	[M+H]^+^	584.2606	585.2679	2.7136	C_33_H_36_N_4_O_6_	Bilirubin^*^	00054
M11	4.94	[M-H]^−^	584.2611	583.2563	1.9716	C_33_H_34_N_4_O_6_	Bilirubin^*^	00054
M12	5.47	[M-H]^−^	354.2396	353.2335	1.2924	C_20_H_34_O_5_	Prostaglandin F2α^^*^^	01139
M13	7.21	[M+H]^+^	517.3142	518.3215	2.1383	C_26_H_48_NO_7_P	LysoPC(18:3)	10388
M14	7.54	[M+H]^+^	519.3276	520.3348	1.3858	C_26_H_50_NO_7_P	LysoPC(18:2)	10386
M15	7.97	[M+H]^+^	569.3455	570.3528	1.9041	C_30_H_52_NO_7_P	LysoPC(22:5)	10403
M16	8.41	[M+H]^+^	302.2234	303.2307	1.8236	C_20_H_30_O_2_	Eicosapentanoic acid	01999
M17	8.41	[M-H]^−^	302.2239	301.2168	1.6305	C_20_H_30_O_2_	Eicosapentanoic acid	01999
M18	8.70	[M+H]^+^	425.3486	426.3559	2.4023	C_25_H_47_NO_4_	11Z-Octadecenylcarnitine	13338
M19	9.58	[M+H]^+^	427.3641	428.3713	2.3509	C_25_H_49_NO_4_	Stearoylcarnitine	00848

* *Identified by authenticated standard; t_R:_ Retention time; VIP, Variable importance in projection; HMDB, Human Metabolome Database*.

#### Analysis of metabolic pathways

To explore potential metabolic pathways, 16 significantly reversed endogenous biomarkers in the ANIT + vehicle group pretreated with high-dose HQD were imported into the MetaboAnalyst software (McGill University, Montreal, Canada). The main metabolic pathways were constructed (Table 3). Primary BA biosynthesis, glutathione metabolism, glutamine and glutamate metabolism, and arginine and proline metabolism were selected as the most important metabolic pathways according to their statistical significance (*p* < 0.05, Table 3).

**Table 3:** Pathway analysis of cholestasis pretreated by HQD.

**Pathway Name**	**Total**	**Hits**	***p***	**−log (*p*)**	**Holm *p***	**FDR**	**Impact**
Primary bile acid biosynthesis	46	3	0.004404	5.4252	0.36113	0.36113	0.05952
Glutathione metabolism	26	2	0.01609	4.1296	1	0.65969	0.01431
D-Glutamine and D-glutamate metabolism	5	1	0.03827	3.2631	1	0.88946	0
Arginine and proline metabolism	44	2	0.043388	3.1376	1	0.88946	0.12736
Taurine and hypotaurine metabolism	8	1	0.060587	2.8037	1	0.92826	0
Nitrogen metabolism	9	1	0.067921	2.6894	1	0.92826	0
Aminoacyl-tRNA biosynthesis	69	2	0.096776	2.3354	1	1	0
Alanine, aspartate and glutamate metabolism	24	1	0.17186	1.7611	1	1	0.14979
Porphyrin and chlorophyll metabolism	27	1	0.19134	1.6537	1	1	0.0415
Cysteine and methionine metabolism	27	1	0.19134	1.6537	1	1	0.08685
Glycerophospholipid metabolism	30	1	0.2104	1.5587	1	1	0.04444
Arachidonic acid metabolism	36	1	0.2473	1.3971	1	1	0
Pyrimidine metabolism	41	1	0.27685	1.2843	1	1	0
Biosynthesis of unsaturated fatty acids	42	1	0.28263	1.2636	1	1	0
Purine metabolism	68	1	0.41896	0.86999	1	1	0

### HQD reverses disordered homeostasis of BA in cholestatic mice

**Figure 3:**
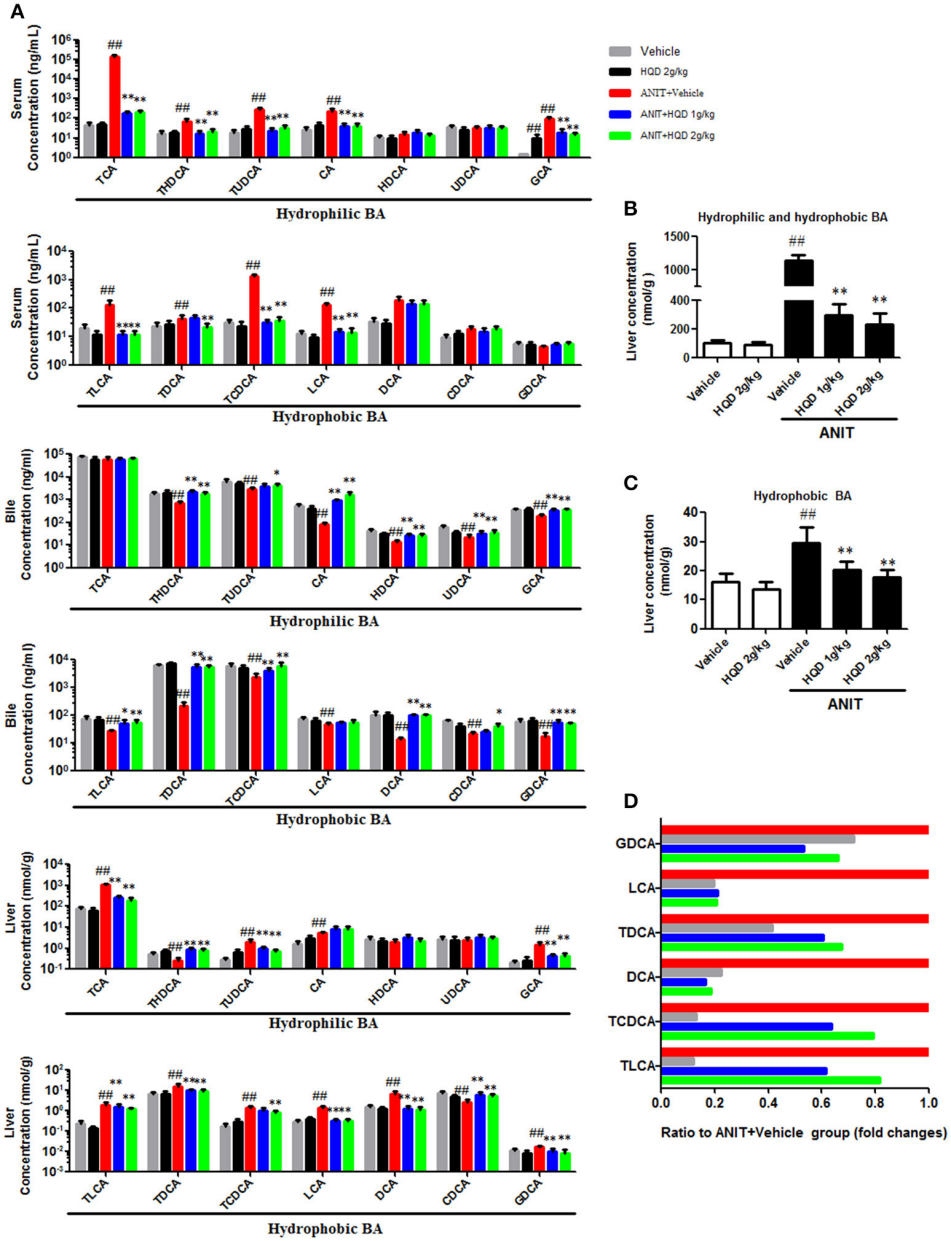
Huangqi decoction (HQD) reverses disordered homeostasis of bile acid (BA) in cholestatic mice. **(A)** Serum, liver, and biliary BA profiling. **(B)** Liver hydrophilic and hydrophobic BA. **(C)** Liver hydrophobic BA. **(D)** Decreased fold changes of six hydrophobic BAs in liver relative to alpha-naphthylisothiocyanate (ANIT)-treated group. Data are means ± *SD, n* = 10. ^##^*p* < 0.01 compared with vehicle-treated group and ^*^p < 0.05 and ^**^p < 0.01 compared with ANIT + Vehicle group. ANIT, alpha-naphthylisothiocyanate; CA, cholic acid; GCA, glycocholic acid; TCA, taurocholic acid; CDCA, chenodeoxycholic acid; GCDCA, glycochenodeoxycholic acid; TCDCA, taurochenodeoxycholic acid; DCA, deoxycholic acid; GDCA, glycodeoxycholic acid; TDCA, taurodeoxycholic acid; UDCA, ursodeoxycholic acid; GUDCA, glycoursodeoxycholic acid; TUDCA, tauroursodeoxycholic acid; LCA, lithocholic acid; GLCA, glycolithocholic acid; TLCA, taurolithocholic acid; HDCA, hyodeoxycholic acid; GHDCA, glycohyodeoxycholic acid; THDCA, taurohyodeoxycholic acid.

As shown in Figure 3, the major hydrophilic BA species such as TCA, THDCA, TUDCA, CA, and GCA, which constituted 98.9% of the serum BA in the ANIT+vehicle group, were significantly reduced by HQD. In addition, serum hydrophobic BAs such as TLCA, TDCA, TCDCA, and LCA markedly increased in the ANIT + vehicle group and were reversed in the ANIT-treated group pretreated with high-dose HQD (Figure 3A). Moreover, compared to levels in the vehicle group, the liver hydrophilic and hydrophobic BAs both increased in the ANIT + vehicle group and were significantly decreased by pretreatment with HQD (Figures 3B,C). LCA and DCA, the two most toxic and the major hydrophobic BA species present in the liver tissue, were significantly decreased by HQD pretreatment (Figure 3D). Notably, biliary hydrophobic and hydrophilic BA secretions decreased in the ANIT + vehicle group compared with that in the vehicle group but were significantly reversed by HQD pretreatment (Figure 3A).

### Effects of HQD on liver BA transporter and metabolic enzyme expression in cholestatic mice

**Figure 4:**
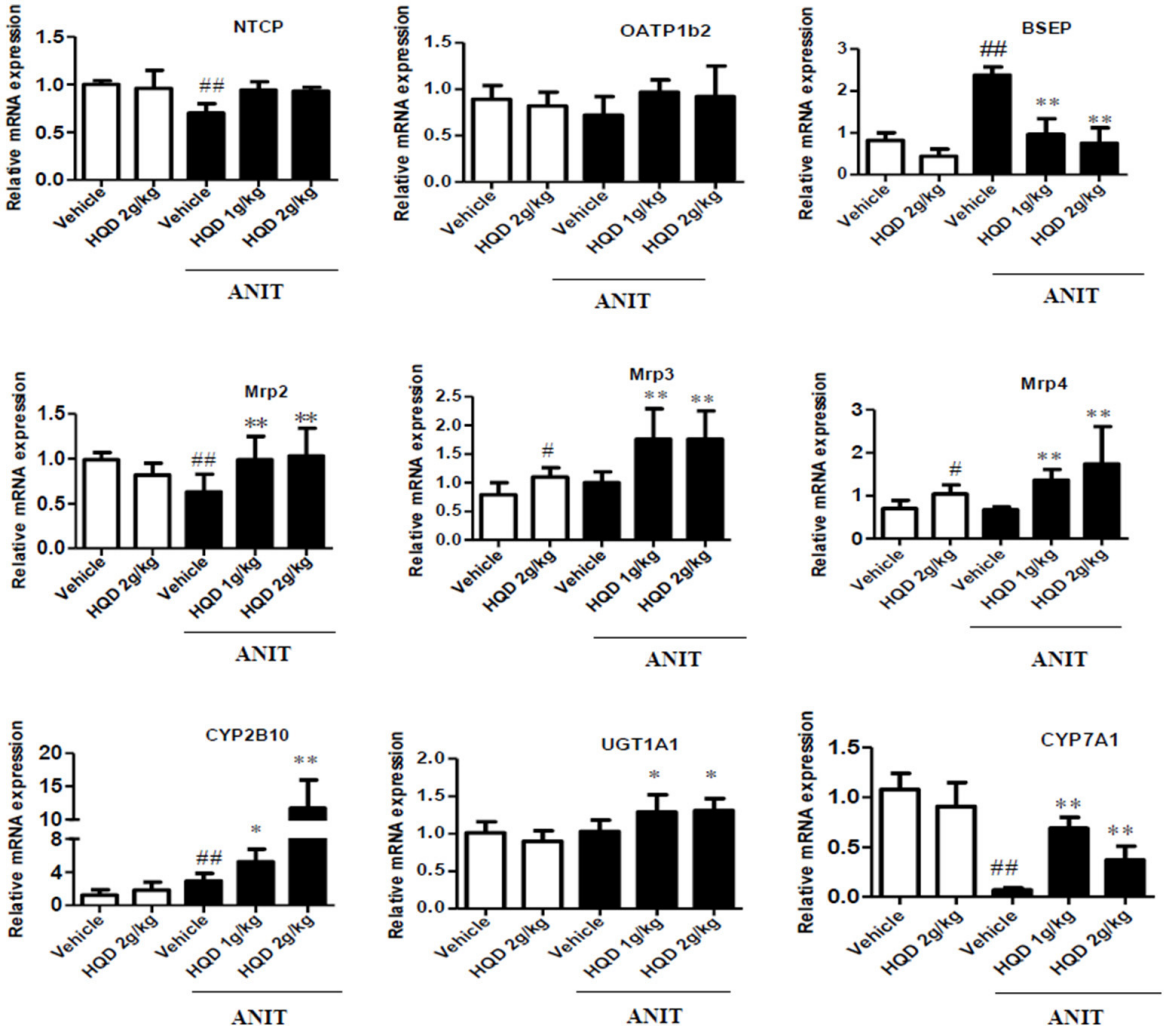
Effects of Huangqi decoction (HQD) on bile acid (BA) transporters and metabolic enzyme mRNA expression levels in cholestatic mouse livers. Data are mean ± *SD, n* = 5. ^#^*p* < 0.05 and ^##^*p* < 0.01 compared with Vehicle group, and ^*^p < 0.05 and ^**^p < 0.01 compared with ANIT + Vehicle group. ANIT, alpha-naphthylisothiocyanate; Mrp, multidrug resistance-associated protein; NTCP, Na^+^/taurocholate cotransporter; OATP1B2, organic anion transporter 1B2; BSEP, bile salt export pump; CYP2B10, cytochrome P450 [Cyp] 2B10; UGT1A1, UDP-glucuronosyltransferase 1A1; CYP7A1, cytochrome P450 [Cyp] 7A1.

**Figure 5:**
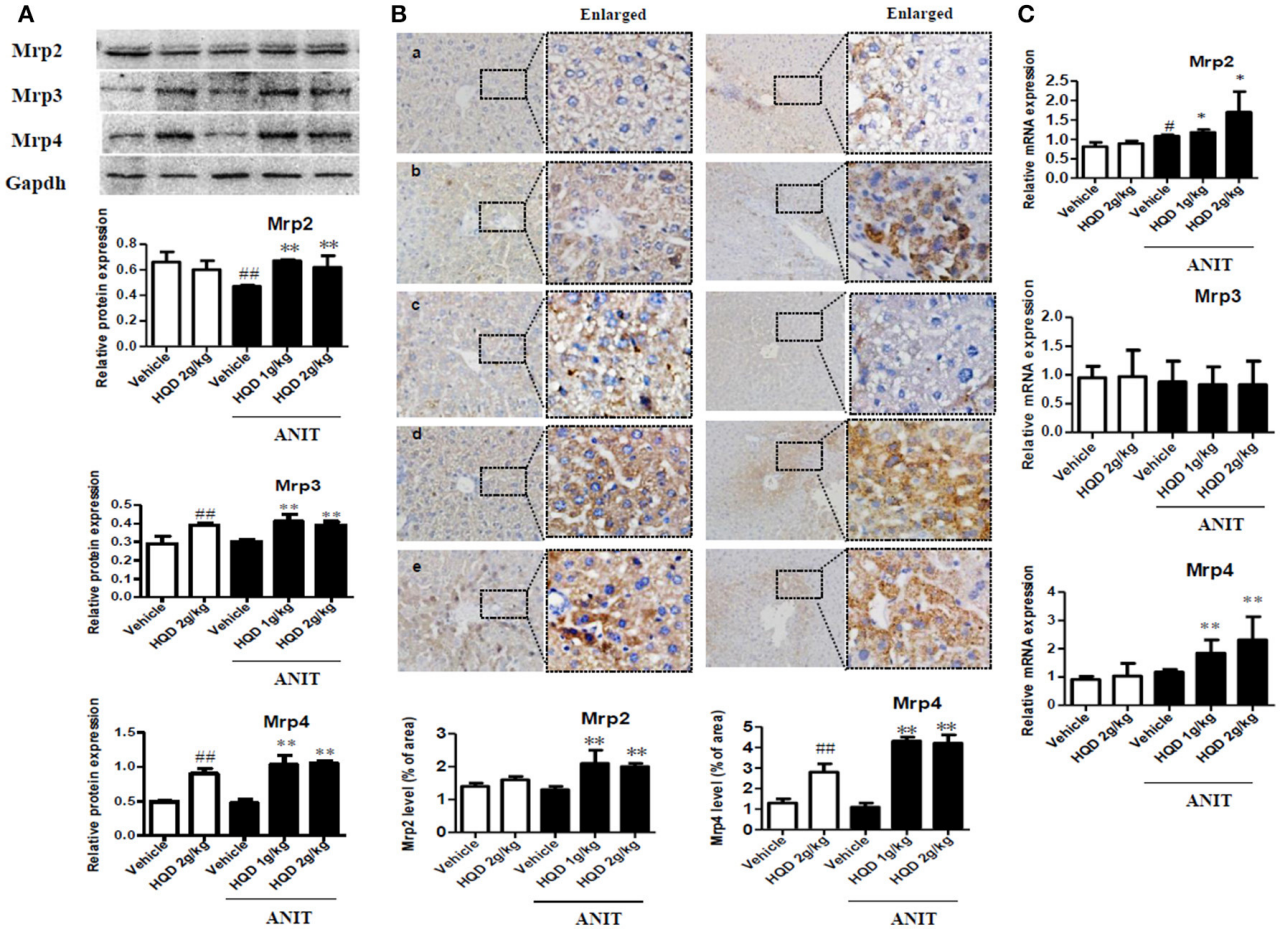
Effects of Huangqi decoction (HQD) on liver and kidney multidrug resistance protein 2 (Mrp2), Mrp3, and Mrp4 transporter expression in cholestatic mice. **(A)** Protein expression of liver efflux transporter Mrps measured using western blotting. **(B)** Quantitative analysis of liver expression levels of Mrp2 and Mrp4 detected using immunohistochemistry. Groups: a, Vehicle; b, HQD 2 g/kg; c, ANIT + Vehicle; d, ANIT + HQD 1 g/kg; e, ANIT + HQD 2 g/kg; **(C)** Kidney Mrp2, Mrp3, and Mrp4 mRNA expression measured using real-time polymerase chain reaction (PCR). Data are means ± *SD, n* = 3 for western blotting and *n* = 5 for immunohistochemistry. ^#^*p* < 0.05, ^##^*p* < 0.01 compared with Vehicle group, and ^*^p < 0.05 and ^**^p < 0.01 compared with ANIT + Vehicle group. ANIT, alpha-naphthylisothiocyanate; Mrp, multidrug resistance-associated protein.

BAs are taken up by the basolateral Na^+^/taurocholate cotransporter (Ntcp) and organic anion transporter 1b2 (Oatp1b2) and are subsequently exported into the bile by the canalicular bile salt export pump (Bsep) and Mrp2 transporters. Mrp3 and Mrp4 transporters are alternative basolateral BA exporters. The mRNA expression of Mrp2 and Ntcp markedly decreased, and that of Bsep and Mrp3 increased in the liver samples of mice in the ANIT + Vehicle group compared to that in the liver of the vehicle group mice. HQD pretreatment significantly reversed the ANIT-induced decrease in Mrp2 to a physiologically normal level (Figure 4). Notably, HQD pretreatment enhanced the mRNA expression of Mrp3 and Mrp4. Consistent with the mRNA expression data, Mrp2, Mrp3, and Mrp4 protein expression levels significantly increased in the ANIT-treated group pretreated with HQD compared with that in the ANIT + Vehicle group (Figure 5A).

The immunohistochemical analysis corroborated the increased protein expression of Mrp2 and Mrp4 (Figure 5B). Metabolizing enzymes that play important roles in BA homeostasis were also investigated. As shown in Figure 4, compared with that in the vehicle group, the mRNA expression of cytochrome P450 (CYP) 7A1 significantly decreased in the ANIT + Vehicle group (Figure 4), whereas HQD pretreatment attenuated the suppression of ANIT-induced CYP7A1 expression. In addition, HQD pretreatment markedly increased the mRNA expression levels of the hepatic metabolizing enzyme CYP2B10 and slightly increased that of UGT1A1 (Figure 4).

### HQD increases urinary BA excretion and expression of kidney efflux transporters

As shown in Supplementary Figure 4, urinary BA excretion significantly increased in the ANIT + vehicle group compared with that in the vehicle group. Interestingly, both the ANIT-treated group pretreated with low- and high-dose HQD showed significantly increased urinary BA excretion (Supplementary Figure 4). Additionally, both HQD pretreatment groups showed a significant increase in the mRNA expression levels of the kidney Mrp2 and Mrp4 (Figure 5C).

### HQD reverses disordered homeostasis of glutathione in cholestatic mice

**Figure 6:**
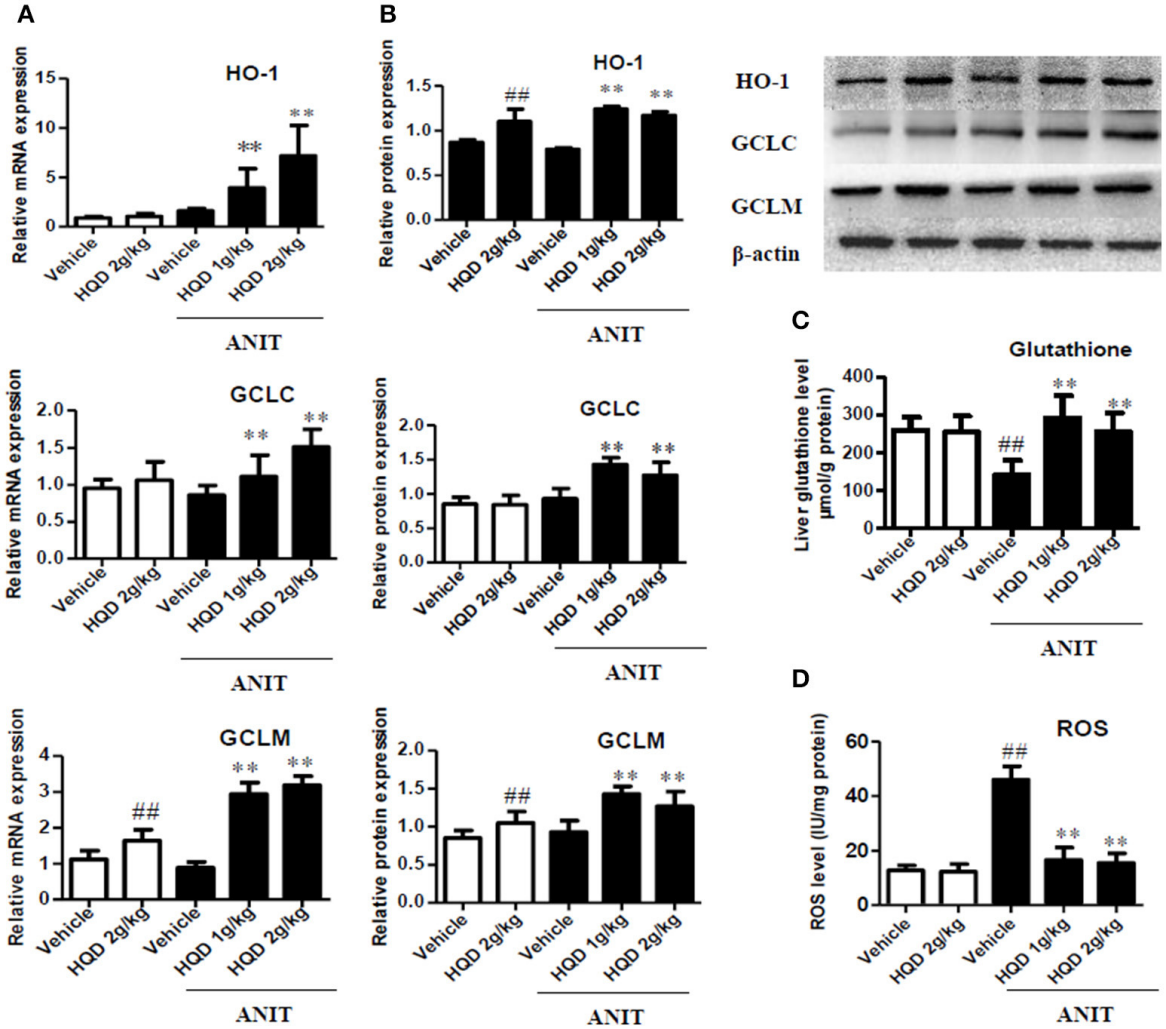
Huangqi decoction (HQD) reverses disordered liver homeostasis of glutathione in mice with intrahepatic cholestasis. **(A)** Liver mRNA expression of HO-1, GCLC, and GCLM measured using real-time polymerase chain reaction (PCR). **(B)** The liver protein expression of HO-1, GCLC, and GCLM measured by western blotting. **(C)** Liver glutathione. **(D)** Liver ROS. The data are expressed as the mean ± *SD, n* = 3 for western blotting and *n* = 5 for real-time PCR. ^##^*p* < 0.01 and ^**^p < 0.01 compared with Vehicle and ANIT + Vehicle groups. ANIT, alpha-naphthylisothiocyanate; HO-1, heme oxygenase-1; GCLC, glutamate-cysteine ligase, catalytic subunit; GCLM, glutamate-cysteine ligase modifier subunit; ROS, reactive oxygen species.

As shown in Figures 6A,B, compared with the ANIT + vehicle group, the ANIT-treated groups pretreated with both low- and high-dose HQD showed significantly increased mRNA and protein expression levels of the hepatic antioxidant enzymes GCLC, GCLM, and HO-1 (Figures 6A,B). In addition, both low- and high-dose HQD pretreatment reversed the ANIT-induced decrease in the hepatic glutathione level (Figure 6C), and decreased the ANIT-induced increase in ROS (Figure 6D).

### HQD increases Nrf2 nuclear translocation and expression of phospho-ERK1/2 and phospho-p38 in cholestatic mice

**Figure 7:**
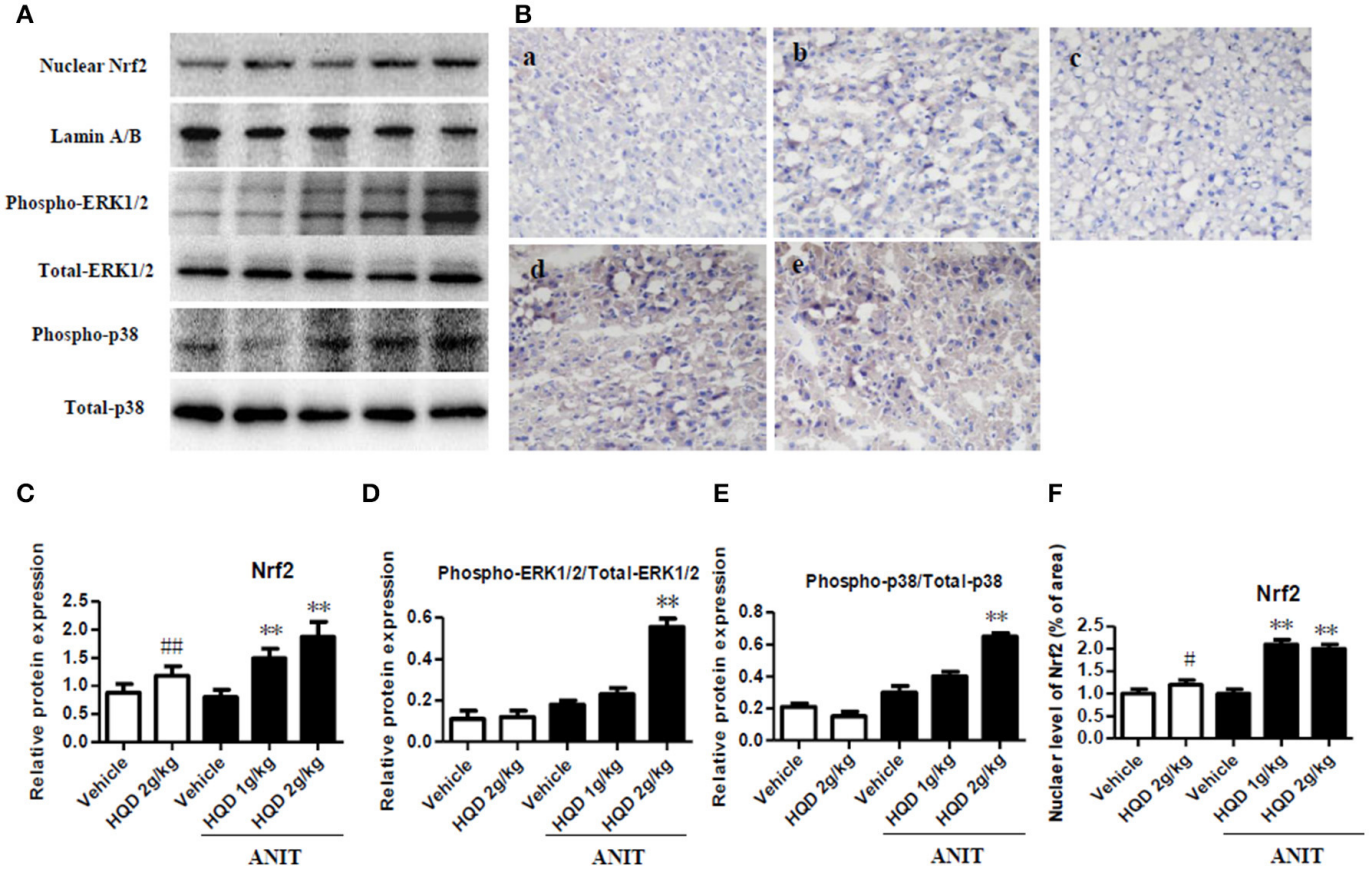
Huangqi decoction increases liver Nrf2 nuclear translocation, and phospho-ERK1/2 and phospho-p38 expression in mice with intrahepatic cholestasis. **(A)** The protein expression of nuclear Nrf2, and phospho-ERK1/2 and phospho-p38 detected by western blot. **(B)** The liver level of nuclear Nrf2 measured using immunohistochemistry. a, Vehicle group; b, HQD 2 g/kg group; c, ANIT+Vehicle group; d, ANIT+HQD 1 g/kg group; e, ANIT+HQD 2 g/kg group **(C)**. Quantitative analysis of nuclear Nrf2 expression. **(D,E)** Quantitative analysis of expression of phospho-ERK1/2 and phospho-p38. **(F)**. Quantitative analysis of nuclear located nuclear factor-E2-related factor-2 (Nrf2) level. Data are means ± *SD*, ^#^*p* < 0.05 and ^##^*p* < 0.01 compared with Vehicle group, and ^**^p < 0.01 compared with ANIT + Vehicle group. ANIT, alpha-naphthylisothiocyanate; ERK1/2, extracellular regulated protein kinase1/2; Nrf2: nuclear factor-E2-related factor-2.

As shown in Figure 7A, both the low- and high-dose HQD pretreatments markedly increased the nuclear Nrf2 levels (Figures 7A,C) compared with levels in the group treated with ANIT alone. In addition, consistent with this result, the low- and high-dose HQD pretreatments also significantly increased the nuclear level of Nrf2 in the livers of cholestatic mice (Figures 7B,F). Moreover, HQD at high dose also significantly induced the activation of phospho-ERK 1/2 and phospho-p38 kinase, which is an upstream molecule of Nrf2, in the livers of cholestatic mice compared with that in the ANIT + vehicle group (Figures 7A,D,E).

## Discussion

HQD, a classic TCM, has hepatoprotective effects (Du et al., 2012; Liu et al., 2012; Zhang G. B. et al., 2015); however, its effect against intrahepatic cholestasis was unknown before this study. In the current study, the experimental conditions were the same for the group treated with ANIT alone and the ANIT + HQD groups except for the HQD pretreatment. However, the ANIT-induced intrahepatic cholestasis and liver injury were significantly ameliorated in the ANIT + HQD groups as indicated by the serum biochemical parameters as well as the morphology and electron microscopy results. Overall, these results demonstrate that HQD protected against intrahepatic cholestasis.

Metabolomics aids in the systematic analysis of metabolites in biological samples, and has been increasingly used to discover and identify biomarkers and perturbed biological pathways. Recent studies have shown that this strategy improves the understanding of metabolic pathways that may elucidate the mechanisms underlying TCM (Xiang et al., 2012; Zhang Z. H. et al., 2015; Ma et al., 2016; Zhao et al., 2016). In the present study, the metabolomic analysis demonstrated that the main metabolic pathways affected by HQD treatment were those involved in primary BA biosynthesis and the metabolism of glutathione, glutamine, and glutamate. Glutamine is converted to glutamate by glutaminase (Tapiero et al., 2002). Glutamate is a precursor of glutathione, which is synthesized by two conjugation reactions catalyzed by GCL and glutathione synthase (Sappington et al., 2016). Therefore, the present results suggest that modulating the homeostasis of BA and glutathione may underlie the protective effects of HQD against intrahepatic cholestasis.

In the current study, we used ANIT, a widely used chemical, to mimic human intrahepatic cholestasis, which is characterized by hepatocyte BA accumulation and disordered BA homeostasis. Disordered homeostasis of BA could further induce liver injury (Pollheimer et al., 2014). In the current study, increased serum and liver BA and decreased bile BA, as measured using UPLC-MS/MS, were found in the ANIT-treated group compared with that in the vehicle group. This result indicates that homeostasis of BA was disordered in the ANIT-induced group. However, HQD treatments decreased the serum and liver BA levels and increased biliary and urinary BA excretion. These observations suggest that the protective effect of HQD against ANIT-induced intrahepatic cholestasis may be associated with its role in modulating the homeostasis of BA, which was in line with the results of the metabolomics study.

Additionally, BA homeostasis is mediated largely by the hepatic phase I and II metabolizing enzymes, and by uptake and efflux transporters (Trauner and Boyer, 2003; Salvatore et al., 2012; Cai et al., 2014; Cuperus et al., 2014; Meng et al., 2015). In this study, HQD increased the expression of the hepatic metabolizing enzymes CYP2B10 and UGT1A1, which catalyze BA hydroxylation and glucuronidation. In addition, HQD increased the expression of the hepatic BA efflux transporters Mrp2, Mrp3, and Mrp4, as well as the renal expression of Mrp2 and Mrp4. These effects explain why HQD increased biliary and urinary BA excretion in the cholestatic mice. Importantly, the increased urinary BA excretion may contribute to decreasing the serum BA concentration. Overall, our results suggest that HQD increased the expression of specific BA metabolizing enzymes and efflux transporters, thereby promoting the detoxification and excretion of hepatotoxic BAs and reversing the disordered BA homeostasis in intrahepatic cholestasis.

Glutathione plays an important role in eliminating free radicals and in oxidative stress defense mechanisms. Obstructive cholestasis can impair hepatic glutathione homeostasis by downregulating its key synthesis enzyme (Neuschwander-Tetri et al., 1996). Therefore, glutathione supplementation can attenuate oxidative stress and decrease obstructive cholestatic liver injury (Chen et al., 2015). However, the role of glutathione homeostasis in intrahepatic cholestasis is not entirely clear. In the current study, HQD restored the decreased hepatic glutathione content and suppressed the increased hepatic ROS generation in the intrahepatic cholestatic mice. These effects suggest that the alleviation of the disordered glutathione homeostasis and decreased the oxidative stress, which are typical in this mouse model. Glutathione is synthesized from amino acids via *two* ATP-dependent enzymatic steps in the cytosol of cells. One of the steps that is considered to be rate-limiting is catalyzed by GCL, which is composed of GCLC and CCLM, the catalytic and modifier subunits, respectively (Neuschwander-Tetri et al., 1996). In this study, we showed that HQD treatment increased the expression levels of GCLC, GCLM, and HO-1, and increased the synthesis of hepatic glutathione in the cholestatic mice. Thus, our results demonstrate that HQD protected against intrahepatic cholestasis by reversing the disordered glutathione homeostasis.

The expression of BA transporters and metabolizing/antioxidant enzymes is tightly regulated by transcription factors (Weerachayaphorn et al., 2012; Li and Chiang, 2013). Nrf2 is one of the key transcription factors that plays an important role in the cellular defense response and regulates the adaptive responses to counteract endogenous and exogenous oxidative stressors (Weerachayaphorn et al., 2012; Suzuki and Yamamoto, 2015). Nrf2 signaling is controlled by Kelch-like ECH-associated protein 1 (Keap1), a substrate adaptor protein for a Cullin3-based E3 ubiquitin ligase that facilitates Nrf2 degradation by a ubiquitin-proteasome pathway. Under basal conditions, Nrf2 is bound to Keap1 in the cytoplasm and is susceptible to proteasomal degradation (Suzuki and Yamamoto, 2015). Following the disruption of the Keap1-Nrf2 complex, Nrf2 is translocated into the nucleus where it binds to the antioxidant response elements (AREs) of promoters to regulate its target genes such as the Mrp efflux transporters and the transcription of GCLC, GCLM, and HO-1 enzymes (Chen et al., 2014; Pang et al., 2015; Sun et al., 2016). In the current study, after treatment with HQD, Nrf2 was subsequently translocated into the nucleus as indicated by the increased level of nuclear Nrf2 in cholestatic mice. Additionally, previous studies have been reported that MAPKs, mainly including ERK1/2 and p38, play important roles in regulating Nrf2 transcriptional activation (Bak et al., 2016; Wong et al., 2016). In the present study, HQD induced the phosphorylation-induced activation of ERK1/2 and p38 and promoted the nuclear translocation of Nrf2 in cholestatic mice. Therefore, the activation of Nrf2 by HQD may also underlie its role in the mitogen-activated protein kinase (MAPK) pathway. In addition to the expression levels of Nrf2, that of the BA transporters and metabolizing enzymes are also regulated by nuclear receptors. In this study, we found that HQD also increased the expression of CAR and its target gene *CYP2B10*. CAR is also regulated by Nrf2 (Wu et al., 2012). Hence, our results suggest that HQD may activate ERK1/2 and p38 and increase the transcriptional activation of hepatic Nrf2, thereby inducing the transcription of downstream target genes of metabolizing enzymes, efflux transporters, and antioxidative enzymes. Furthermore, these effects reversed the disordered homeostasis of BAs and glutathione.

In the present study, the liver expression levels of Nrf2 and its target genes such as *GCLC, GCLM*, and *HO-1* were also much higher in the ANIT + HQD groups than they were in the HQD group. This effect may be associated with the different effects of HQD in cholestatic mice and normal mice. Previous studies also have been reported that drugs such as oleanolic acid or baicalin induced higher Nrf2 expression in cholestatic mice than they did in normal mice (Chen et al., 2014; Shen et al., 2017). This effect may be associated with the different physiological states of the animals and would require further investigation.

The ANIT-induced increase in the mRNA expression levels of the efflux transporter Bsep and the metabolizing enzymes Cyp3a11 and Sult2a1 (Supplementary Figure 5) may indicate self-adaptive responses that protect against the excessive hepatic accumulation of toxic BAs (Liu et al., 2013). However, this response did not prevent the progression of cholestatic liver disease. HQD restored the expression of Bsep and Sult2a1 but did not affect Cyp3a11. This might be attributed to the HQD-mediated decrease in the hepatic accumulation of toxic BAs.

TCM usually consist of multiple herbs with numerous ingredients, which leads to uncertainty and complexity in the attempts to perform dose-effect analyses (Xie et al., 2011). HQD is a traditional Chinese herbal decoction consisting of multiple ingredients. In the current study, HQD did not show dose-dependency, although high-dose HQD showed a stronger trend than that of the low dose in some efficacy indexes (AST, TBIL, TBA, CYP2B10, HO-1, and Nrf2) in cholestatic mice. Additionally, it is important to perform QC analysis of TCMs to ensure that the biological effects of various batches remain consistent. In our study, a previously described UHPLC-MS/MS method was used to perform the QC analysis of HQD (Luo et al., 2014). Triterpenoid saponins and flavonoids, which are the main compounds in HQD, include astragaloside, glycyrrhizic acid, and isoliquiritigenin that have various pharmacological effects including hepatoprotective and antioxidative (Guo et al., 2013; Li et al., 2013; Jin et al., 2014). Previous studies have shown that the antioxidative effects of these triterpenoid saponins stimulate the expression of hepatic Nrf2 (Guo et al., 2013; Li et al., 2013; Jin et al., 2014). Therefore, they may be the pharmacologically active compounds of HQD responsible for the protection against intrahepatic cholestasis. However, the effects of the active ingredients of HQD on intrahepatic cholestasis need further investigation.

The protective effect of HQD against intrahepatic cholestatic liver injury was unknown before this study. In the current study, the ANIT-induced cholestatic liver injury model was used, which is an established method that was useful for understanding the protective effect of HQD on cholestatic liver disease. However, using ANIT at a lower concentration in acute or subacute experiments to further investigate the activity of HQD would also be expedient in future research studies.

HQD, a classic herbal medicine, has been used in clinic for 1,000 years in China. In the current study, we found that HQD provides a well protection against ANIT-induced cholestatic mice, which suggested that HQD may be an effective therapeutic strategy in treating clinical liver disease patients with intrahepatic cholestasis. Additionally, from the study above, HQD also exerted a better protective efficiency than UDCA. There have shown that some cholestatic patients that are not responded to UDCA (Beuers et al., 2015; Ali et al., 2016). Therefore, HQD may be also used to treat those patients.

In conclusion, our results indicate that HQD may protect against intrahepatic cholestasis by increasing the efflux transporters and antioxidative enzymes, as well as reversing disordered homeostasis of BAs and glutathione.

## Author contributions

J-SW, Y-FL, and Y-YL performed the experiments, analyzed the data, and wrote the manuscript. YD, W-KL, MZ, Z-CS, RS, T-MW, and B-LM collected and prepared samples. Y-FL, Y-YL, MZ, and Z-CS performed the analyses. PL amended the paper. J-SW and Y-MM designed the study and amended the paper.

### Conflict of interest statement

The authors declare that the research was conducted in the absence of any commercial or financial relationships that could be construed as a potential conflict of interest.
