# Supplementary material for: Huangqi Decoction Alleviates Alpha-Naphthylisothiocyanate Induced Intrahepatic Cholestasis by Reversing Disordered Bile Acid and Glutathione Homeostasis in Mice
Source: Front Pharmacol. 2017 Dec 21;8:938. doi: 10.3389/fphar.2017.00938 (PMC5742571; doi:10.3389/fphar.2017.00938)
Supplement: Supplementary file 1 [file Presentation1.pdf]

Huangqi Decoction Alleviates Alpha-Naphthylisothiocyanate Induced Intrahepatic Cholestasis by Reversing Disordered Bile Acid and Glutathione Homeostasis in Mice

Jia-Sheng Wu[1], Yi-Fei Li[1], Yuan-Yuan Li[1], Yan Dai[1], Wen-Kai Li[1], Min Zheng[1], Zheng-Chun Shi[1], Rong Shi[1], Tian-Ming Wang[1], Bing-Liang Ma[1], Ping Liu[2], Yue-Ming Ma[1, 3 *]

[1]Department of Pharmacology, School of Pharmacy, Shanghai University of Traditional Chinese Medicine, Shanghai 201203, China

[2]Key Laboratory of Liver and Kidney Diseases (Ministry of Education), Institute of Liver Diseases, Shuguang Hospital, Shanghai University of Traditional Chinese Medicine, 528 Zhangheng Road, Shanghai 201204, China

[3]Shanghai Key Laboratory of Compound Chinese Medicines, Shanghai University of Traditional Chinese Medicine, Shanghai 201203, China

[*]**Corresponding author:**

Prof. Yue-Ming Ma,

Department of Pharmacology, School of Pharmacy, Shanghai University of Traditional Chinese Medicine, 1200 Cailun Road, Shanghai 201203, China

Email: mayueming@ shutcmedu.cn,

Phone: 86-21-51322386,

Fax: 86-21-51322386

Supplementary methods

1. Serum metabolomic analysis

Data processing and analysis, identification of potential biomarkers were performed as previously described [1].

Data processingand analysis

The raw ultra-pressure liquid chromatography coupled with mass spectrometry (UPLC-MS/MS) data were processed using the SIEVE 2.1 software (Thermo Fisher Scientific, USA) for peak deconvolution and alignment. The method parameters were as follows: ion m/z tolerance (10 ppm), ion retention time tolerance (0.2 min), ion intensity threshold (5’000 counts), and retention time range: 0-16 min. The mass-to-charge ratio range is 50-1000. After completing the integration parameters, a report of peaks based on areas, retention time and *m/z* was generated for each sample. These screened data were processed according to the “80 % rule”. After normalization, variables with relative standard deviation (RSD) lower than 30 % in quality control (QC) samples were chosen. Pareto transformation was performed to stabilize the variance throughout the intensity range. The final preprocessed data table was processed using the SIMCA-P software version 14.0 (Umetrics; Malmö, Sweden) for perform principal components analysis (PCA) and orthogonal partial least squares discriminant analysis (OPLS-DA). The corresponding variable importance (VIP value) was calculated in the OPLS-DA model. Then, the nonparametric Kruskal–Wallis rank sum test was performed to determine the significance of each variable, and the relevant false discovery rates based on the *p* value were estimated in the context of multiple testing. A potential metabolic biomarker was selected when the VIP value was more than 1.0 and *p* value was less than 0.05.

Identification of potential biomarkers

Identification of the potential biomarkers was achieved through a mass-based search

followed by manual verification. First, the m/z value of the molecular ion of interest was searched against four databases: the Human Metabolome Database (HMDB), METLIN, KEGG PATHWAY Database, and LIPID MAPS. Then, the putative identifications were verified by comparing the MS^n fragmentation patterns and retention time with those of authentic standard compounds.

2. Bile acid profiling measured by UPLC-MS/MS

2.1 Sample preparation

Bile and serum were diluted 200-fold and 5-foldwith water, respectively. The diluted bile or serum sample was mixed with 150 μL of methanolfor protein precipitation, to which 0.32 ng/μL of theinternal standard was added, followed by 1 min of vortex mixing. The samples were centrifuged at 16,000 rpm for 10 minat 4 ℃.A 5-μLaliquot of the supernatant was injected into the UPLC-MS/MS system for analysis. For liver tissue, 50 mg of tissue were homogenized with 900 μL of cold water/methanol (50:50) using an ultrasonicator, followed by centrifugation at 12,000 rpm for 10 min at 4 ℃. The supernatant (50 μL) was added to a 150-μL aliquot of methanol containing the internal standard. After vortex-mixing for 1 min and centrifugation at 12,000 rpm for 10 min at 4 ℃, a5-μL aliquot of the supernatant was injected into the UPLC–MS/MS system.

2.2 Preparation of thereference standard samples

Aliquots of each of the 18reference standard stock solutions of each bile acid(BA) werecombined to obtain a mixed stock solution. Calibration solutions containing all 18 BA reference standards were prepared at a concentration series range of0.5–10’000/5’000 ng/mL in naive pooled serum, bile, or liver tissue depleted of BAs using activated charcoal. The calibration curve and the corresponding regression coefficients were obtained by internal standard adjustment (Supplementary Table S1–S3). All BAs were linear over the measured range.

2.3 UPLC-MS/MS analysis

Serum, liver, and biliary BAs were measured as describedpreviously[2], with minor modification. Briefly, a Waters ACQUITY UPLC system equipped with a binary solvent delivery manager and a sample manager (Waters,Co.; Milford, MA, USA) was used. The mass spectrometer was a Waters Triple-Quadrupole 5500 instrument with an ESI source (Waters). The entire LC−MS system was controlled by the AnaLyst 1.5 software (Waters, Co.).

All chromatographic separations were performed with an ACQUITY BEH C18 column (1.7 μm, 100 mm × 2.1 mm internal dimensions) (Waters, Co.). The mobile phase consisted of 0.1% formic acid in LC−MS-grade water (mobile phase A) and 0.1% formic acid in LC−MS-grade acetonitrile (mobile phase B). The flow rate was 0.3 mL/min with the following mobile phase gradient: 0–1 min (55% B), 1–9 min (55−80% B), 9–11.4 min (80–90% B), 11.4-14.1 min (90–55% B), and 14.1–17 min (55% B). The column was maintained at 45 ℃ and the injection volume of all samples was 5 μL. The mass spectrometer was operated with a 2.8 kV capillary voltage. The source and desolvation gas temperature was 120 and 350 ℃, respectively. The data were collected using multiple reaction monitoring (MRM).

2.4 Data analysis

The raw UPLC-MS/MS data were analyzed using the AnaLyst 1.5 Software (Waters, Co.) to obtain calibration equations and the quantitative concentration of each BA in the samples.Differences in the BA measurements between the groups were analyzed using a Student's *t*-test with p <0.05 considered significant. The regression equation was derived from the standard curve, the relative deviation of the samples' lower limit of quantification(LLOQ) was≤20%, the relative deviation of the other concentrations and quality control (QC)was ≤15%, and the square of the correlation coefficient $r^2$was<0.99. The content of each test sample was calculated from the standard curve.

Supplementary Reference

1. Huang, Y. *et al.* Discovery of safety biomarkers for realgar in rat urine using UFLC-IT-TOF/MS and 1H NMR based metabolomics. *Anal bioanal chem* **405**, 4811-4822 (2013).

2. Yang, L. *et al.* Bile acids metabonomic study on the CCl4- and alpha-naphthylisothiocyanate-induced animal models: quantitative analysis of 22 bile acids by ultraperformance liquid chromatography-mass spectrometry. *Chem Res Toxicol* **21**, 2280-2288 (2008)

***Supplementary*Table 1** Calibration curves of the 18 bile acid in serum

No.	Bile acid	Calibration curve	r2	Linear range (ng /mL)
1	TUDCA	y=0.00033x+0.000831	0.9962	5-10000
2	TCDCA	y=0.0000731x+0.000452	0.9989	5-10000
3	THDCA	y=0.000534x+0.000441	0.9975	5-10000
4	TDCA	y=0.000499x+0.00195	0.9971	5-10000
5	TCA	y=0.000291x+0.000219	0.9994	5-10000
6	TLCA	y=0.000225x+0.000733	0.9966	5-10000
7	UDCA	y=0.00381x+0.00295	0.9990	5-10000
8	CDCA	y=0.000761x+0.000214	0.9979	5-10000
9	HDCA	y=0.000872x+0.000985	0.9990	5-10000l
10	DCA	y=0.00153x+0.00209	0.9981	5-10000
11	CA	y=0.000923x+0.000898	0.9975	5-10000
12	LCA	y=0.000341x+0.0117	0.9989	5-10000
13	GUDCA	y=0.000794x+0.000656	0.9977	5-10000
14	GCDCA	y=0.00013x+0.00004	0.9959	5-10000
15	GDCA	y=0.000217x-0.000164	0.9971	5-10000
16	GCA	y=0.000285x+0.000353	0.9983	5-10000
17	GHDCA	y=0.000542x+0.00122	0.9974	5-10000
18	GLCA	y=0.000152x-0.0000692	0.9924	5-10000

Supplementary**Table 2** Calibration curves of the 18 bile acid in liver

No.	Bile acid	Calibration curve	r2	Linear range (μg /mL)
1	TUDCA	y=0.000532x+0.000675	0.9954	1-5000
2	TDCA	y=0.000672x+0.000672	0.9967	1-5000
3	TCDCA	y=0.000523x+0.000523	0.9937	1-5000
4	TCA	y=0.000414x+0.000414	0.9952	1-5000
5	GCA	y=0.000482x+0.0000309	0.9933	1-5000
6	GCDCA	y=0.000475x+0.0000963	0.9938	1-5000
7	HDCA	y=0.00158x+0.000849	0.9952	1-5000
8	CA	y=0.00391x+0.00175	0.9940	1-5000
9	DCA	y=0.00595x+0.00525	0.9937	1-5000
10	CDCA	y=0.00154x+0.00854	0.9954	1-5000
11	UDCA	y=0.00165x+0.000354	0.9941	1-5000
12	GDCA	y=0.000637x+0.000228	0.9973	1-5000
13	TLCA	y=0.000655x+0.00248	0.9967	1-5000
14	THDCA	y=0.00049x+0.000444	0.9972	1-5000
15	LCA	y=0.00135x+0.112	0.9958	1-5000
16	GUDCA	y=0.0008x+0.000229	0.9959	1-5000
17	GLCA	y=0.000528x+0.0000177	0.9949	1-5000
18	GHDCA	y=0.000882x-0.00000111	0.9956	1-5000

Supplementary**Table 3** Calibration curves of the 18 bile acid in bile

No.	Bile acid	Calibration curve	r2	Linear range (μg /mL)
1	TUDCA	y=0.000538x+0.000312	0.9974	5-10000
2	TDCA	y=0.000442x+0.000376	0.9988	5-10000
3	TCDCA	y=0.0000521x+0.000166	0.9973	5-10000
4	TCA	y=0.000458x+0.000343	0.9978	5-10000
5	GCA	y=0.000264x-0.000139	0.9965	5-10000
6	GCDCA	y=0.000348x-0.000114	0.9992	5-10000
7	HDCA	y=0.00146x-0.0000481	0.9984	5-10000
8	CA	y=0.00207x+0.0000409	0.9970	5-10000
9	DCA	y=0.00247x+0.00302	0.9974	5-10000
10	CDCA	y=0.00153x+0.00207	0.9974	5-10000
11	UDCA	y=0.00272x-0.000045	0.9976	5-10000
12	GDCA	y=0.000396x-0.000187	0.9968	5-10000
13	TLCA	y=0.000678x-0.000379	0.9954	5-10000
14	THDCA	y=0.000554x+0.000136	0.9980	5-10000
15	LCA	y=0.000841x+0.0162	0.9969	5-10000
16	GUDCA	y=0.000764x-0.000267	0.9986	5-10000
17	GLCA	y=0.000208x-0.000121	0.9977	5-10000
18	GHDCA	y=0.000545x-0.000583	0.9972	5-10000

Supplementary**Table 4** Primers used in this study (Real-time PCR)

Gene Name	GenBank Accession	Specise	Primer sequence (5'-3')
Gapdh	NM_008084.3	Mouse	Forward primer AGGTCGGTGTGAACGGATTTG
			Reverse primer GGGGTCGTTGATGGCAACA
Ntcp	NM_001177561.1	Mouse	Forward primer CAAACCTCAGAAGGACCAAACA
			Reverse primer GTAGGAGGATTATTCCCGTTGTG
Oatp1b2	NM_020495.1	Mouse	Forward primer GCACTGCGATGGATTCAGGAT
			Reverse primer AGCTTTGGTCGGTGTAGCTTG
Mrp2	NM_013806.2	Mouse	Forward primer GTGTGGATTCCCTTGGGCTTT
			Reverse primer CACAACGAACACCTGCTTGG
Mrp3	NM_029600.3	Mouse	Forward primer CTGGGTCCCCTGCATCTAC
			Reverse primer GCCGTCTTGAGCCTGGATAAC
Mrp4	NM_001033336.3	Mouse	Forward primer CATCGCGGTAACCGTCCTC
			Reverse primer CCGCAGTTTTACTCCGCAG
Cyp7a1	NM_007824.2	Mouse	Forward primer GAACCTCCTTTGGACAACGGG
			Reverse primer GGAGTTTGTGATGAAGTGGACAT
Bsep	NM_021022.3	Mouse	Forward primer TCTGACTCAGTGATTCTTCGCA
			Reverse primer CCCATAAACATCAGCCAGTTGT
Cyp2b10	NM_009999.4	Mouse	Forward primer TGCTGTCGTTGAGCCAACC
			Reverse primer CCACTAAACATTGGGCTTCCT
Cyp3a11	NM_007818.3	Mouse	Forward primer GGATGAGATCGATGAGGCTCTG
			Reverse primer CAGGTATTCCATCTCCATCACAGT
Ugt1a1	NM_201645.2	Mouse	Forward primer GCTTCTTCCGTACCTTCTGTTG
			Reverse primer GCTGCTGAATAACTCCAAGCAT
Sult2a1	NM_001111296.2	Mouse	Forward primer GAAGGCATACCTTTTCCTGCCAT
			Reverse primer GTAACCAGACACAAGAATATCTCT
HO-1	NM_010442.2	Mouse	Forward primer CCTCACTGGCAGGAAATCATC
			Reverse primer CCTCGTGGAGACGCTTTACATA
GCLC	NM_010295.2	Mouse	Forward primer AGGTTGACGAGAACATGAAAGTGGC
			Reverse primer CCGCCTTTGCAGATGTCTTTCCTGA
GCLM	NM_008129.4	Mouse	Forward primer AATCAGCCCTGATTTGGTCAGG
			Reverse primer CCAGCTGTGCAACTCCAAGGAC
CAR	NM_001243062.1	Mouse	Forward primer TTCAAGCCTCCGGCCTATCT
			Reverse primer TGATCTGTTGCACCATAAACGTG

Supplementary **Table 5** Identification results of serum endogenous metabolites between Vehicle group and ANIT alone treated group

No.	Ta (min)	Molecular ion	Compound MW	Measured Mass (Da)	VIP	Formula	Metabolites
**M1**	0.78	**$[M+H]^+$**	132.0893	133.0965	2.0105	$C_5H_{12}N_2O_2$	Ornithine
**M2**	0.84	**$[M-H]^-$**	146.0694	145.0621	1.2415	$C_5H_{10}N_2O_3$	Glutamine
**M3**	0.95	**$[M+H]^+$**	149.0503	150.0576	2.0454	$C_5H_{11}NO_2S$	Methionine
**M4**	0.97	**$[M+H]^+$**	129.0422	130.0494	1.9262	$C_5H_7NO_3$	Pyroglutamic acid
**M5**	0.97	**$[M+OH]^-$**	129.0427	146.0457	1.2672	$C_5H_7NO_3$	Pyroglutamic acid
**M6**	1.73	**$[M-H]^-$**	118.0630	117.0558	1.2539	$C_5H_{10}O_3$	3-Hydroxyisovaleric acid
**M7**	2.91	**$[M-H]^-$**	179.0579	178.0509	1.0299	$C_9H_9NO_3$	Hippuric acid
**M8**	3.03	**$[M-H]^-$**	130.0629	129.0558	1.0134	$C_6H_{10}O_3$	Ketoleucine
**M9**	3.58	**$[M+H]^+$**	205.0730	206.0803	1.3298	$C_{11}H_{11}NO_3$	Indolelactic acid
**M10**	3.77	**$[M+H]^+$**	515.2887	516.2960	4.1002	$C_{26}H_{45}NO_7S$	Taurocholic acid
**M11**	3.99	**$[M-H]^-$**	499.2952	498.2894	2.1877	$C_{26}H_{45}NO_6S$	Tauroursodeoxycholic acid
**M12**	4.38	**$[M+H]^+$**	189.0781	190.0854	1.1568	$C_{11}H_{11}NO_2$	3-Indolepropionic acid
**M13**	4.58	**$[M-H]^-$**	408.2865	407.2795	2.0064	$C_{24}H_{40}O_5$	Cholic acid
**M14**	4.84	**$[M-H]^-$**	465.3075	464.3009	3.2533	$C_{26}H_{43}NO_6$	Glycocholic acid
**M15**	4.93	**$[M+H]^+$**	584.2606	585.2679	2.7136	$C_{33}H_{36}N_4O_6$	Bilirubin
**M16**	4.94	**$[M-H]^-$**	584.2611	583.2563	1.9716	$C_{33}H_{34}N_4O_6$	Bilirubin
**M17**	5.37	**$[M+H]^+$**	315.2394	316.2466	1.1226	$C_{17}H_{33}NO_4$	Decanoylcarnitine
**M18**	5.47	**$[M-H]^-$**	354.2396	353.2335	1.2924	$C_{20}H_{34}O_5$	Prostaglandin F2α
**M19**	6.36	**$[M-H]^-$**	216.1722	215.1652	3.0107	$C_{12}H_{24}O_3$	3-Hydroxydodecanoic acid
**M20**	7.21	**$[M+H]^+$**	517.3142	518.3215	2.1383	$C_{26}H_{48}NO_7P$	LysoPC(18:3)
**M21**	7.54	**$[M+H]^+$**	519.3276	520.3348	1.3858	$C_{26}H_{50}NO_7P$	LysoPC(18:2)
**M22**	7.97	**$[M+H]^+$**	569.3455	570.3528	1.9041	$C_{30}H_{52}NO_7P$	LysoPC(22:5)
**M23**	8.41	**$[M+H]^+$**	302.2234	303.2307	1.8236	$C_{20}H_{30}O_2$	Eicosapentanoic acid
**M24**	8.41	**$[M-H]^-$**	302.2239	301.2168	1.6305	$C_{20}H_{30}O_2$	Eicosapentanoic acid
**M25**	8.70	**$[M+H]^+$**	425.3486	426.3559	2.4023	$C_{25}H_{47}NO_4$	11Z-Octadecenylcarnitine
**M26**	8.90	**$[M+H]^+$**	547.3614	548.3687	2.0824	$C_{28}H_{54}NO_7P$	LysoPC(20:2)
**M27**	9.58	**$[M+H]^+$**	427.3641	428.3713	2.3509	$C_{25}H_{49}NO_4$	Stearoylcarnitine
**M28**	9.95	**$[M+H]^+$**	549.3767	550.3840	1.9832	$C_{28}H_{56}NO_7P$	LysoPC(20:1)
**M29**	11.16	**$[M+H]^+$**	330.2546	331.2619	1.1972	$C_{22}H_{34}O_2$	Docosapentaenoic acid
**M30**	11.59	**$[M+H]^+$**	551.3925	552.3998	2.7123	$C_{28}H_{58}NO_7P$	LysoPC(20:0)

Supplementary Figure 1

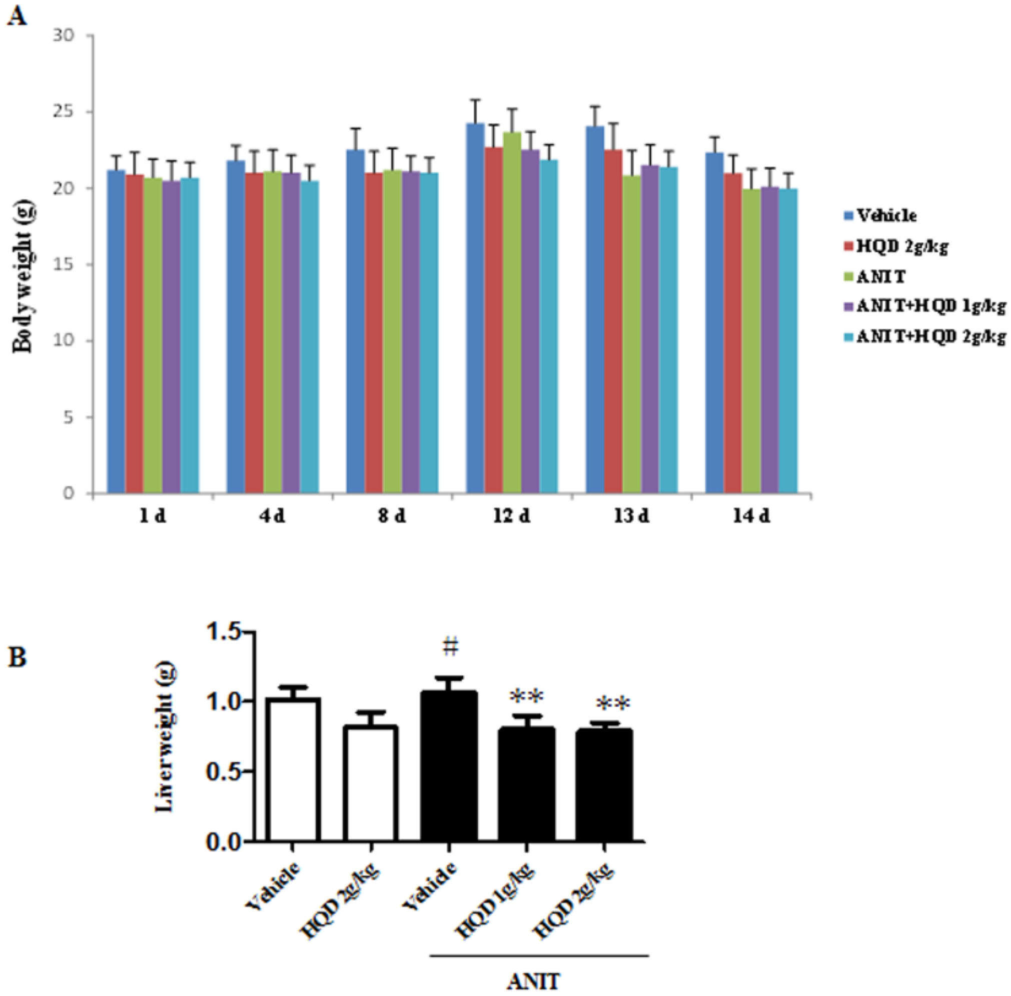

Supplementary Figure 2

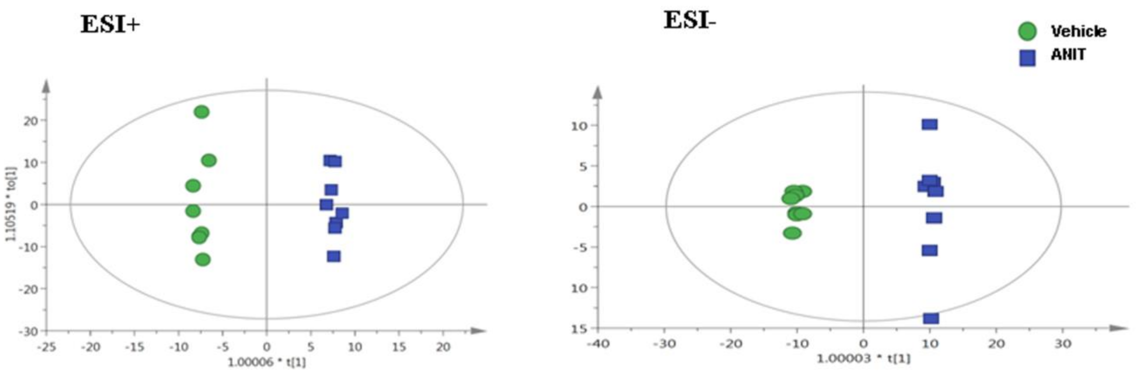

Supplementary Figure 3

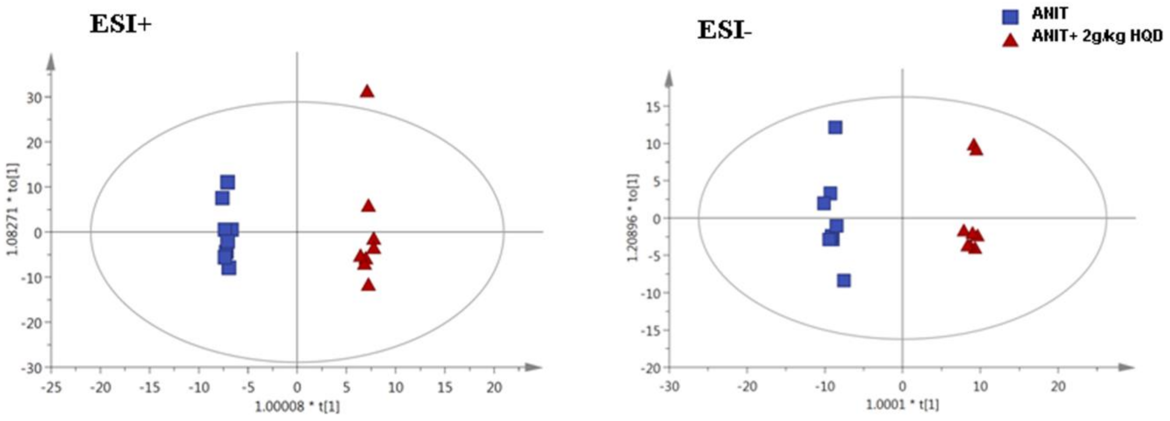

Supplementary Figure 4

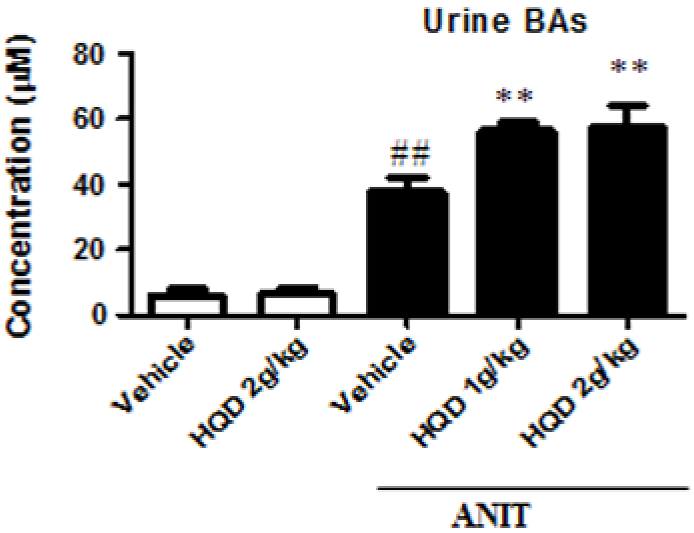

Supplementary Figure 5

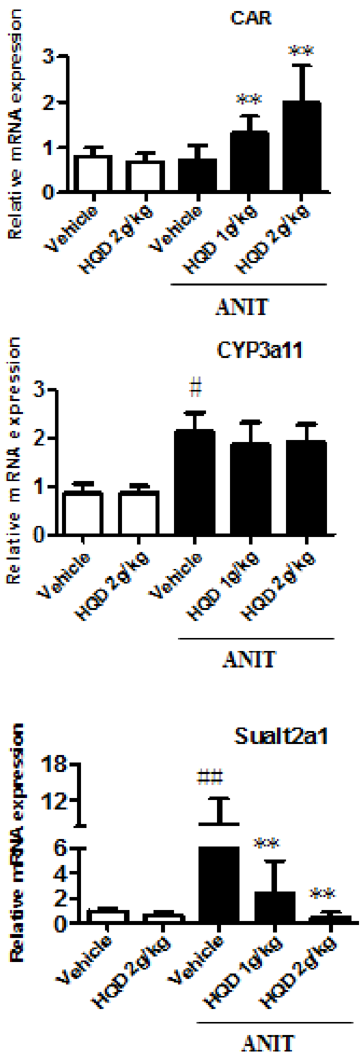

Supplementary figure legends

Supplementary Figure 1. The effects of HQD on body weight and liver weight in mice.

(A) Body weight; (B) Liver weight. Mean ± SD, n = 10.Compared with Vehicle group, $^{\#}p < 0.05$; compared with ANIT alone treated group, $^{*}p < 0.05$ and $^{**}p < 0.01$.d: day; ANIT, alpha-naphthylisothiocyanate

Supplementary Figure2. OPLS-DA scores plot between Vehicle group and ANIT alone treated group

(R2X= 0.899,R2Y=0.995,Q2= 0.950) in ESI+ mode

(R2X= 0.824,Q2Y= 0.997,Q2= 0.968) in ESI- mode

ANIT, alpha-naphthylisothiocyanate

Supplementary Figure3. OPLS-DA scores plot between ANIT alone treated group and ANIT-induced group pretreated with high dose of HQD

(R2X= 0.897,R2Y=0.998,Q2= 0.943) in ESI+ mode

(R2X= 0.787,Q2Y= 0.995,Q2= 0.947) in ESI- mode.

ANIT, alpha-naphthylisothiocyanate

Supplementary Figure 4. The effect of HQD on the level of urine bile acids

The data are expressed as the mean ± S.D., n=10.Compared with the vehicle-treated group, $^{\#\#}p < 0.01$; compared with the ANIT-treated group, $^{*}p < 0.05$ and $^{**}p < 0.01$.ANIT, alpha-naphthylisothiocyanate

Supplementary Figure5. Effects of HQD on liver metabolic enzyme Cyp3a11, Sualt2a1, and nuclear receptor CAR mRNA expression

mean ± SD, n = 5.Compared with Vehicle group, $^{\#\#}p < 0.01$; compared with ANIT alone treated group$^{*}p < 0.05$ and $^{**}p < 0.01$. ANIT, alpha-naphthylisothiocyanate
